# Epigenetic regulation of NK cell differentiation and effector functions

**DOI:** 10.3389/fimmu.2013.00055

**Published:** 2013-02-28

**Authors:** Frank Cichocki, Jeffrey S. Miller, Stephen K. Anderson, Yenan T. Bryceson

**Affiliations:** ^1^Department of Medicine, Center for Infectious Medicine, Karolinska Institute, Karolinska University Hospital HuddingeStockholm, Sweden; ^2^Adult Division of Hematology, Oncology and Transplantation, University of Minnesota Cancer CenterMinneapolis, MN, USA; ^3^Laboratory of Experimental Immunology, Cancer and Inflammation Program, SAIC-Frederick, Inc., National Cancer InstituteFrederick, MD, USA

**Keywords:** NK cell, epigenetics, transcription factors, development, memory

## Abstract

Upon maturation, natural killer (NK) cells acquire effector functions and regulatory receptors. New insights suggest a considerable functional heterogeneity and dynamic regulation of receptor expression in mature human NK cell subsets based on different developmental axes. Such processes include acquisition of lytic granules as well as regulation of cytokine production in response to exogenous cytokine stimulation or target cell interactions. One axis is regulated by expression of inhibitory receptors for self-MHC class I molecules, whereas other axes are less well defined but likely are driven by different activating receptor engagements or cytokines. Moreover, the recent identification of long-lived NK cell subsets in mice that are able to expand and respond rapidly following a secondary viral challenge suggest previously unappreciated plasticity in the programming of NK cell differentiation. Here, we review advances in our understanding of mature NK cell development and plasticity with regards to regulation of cellular function. Furthermore, we highlight some of the major questions that remain pertaining to the epigenetic changes that underlie the differentiation and functional specialization of NK cells and the regulation of their responses.

## Introduction

Epigenetics refers to functionally relevant modifications to the genome that do not involve a change in the nucleotide sequence, but may impact gene expression, cellular phenotype, and function. Examples of such modifications are acetylation, methylation or phosphorylation of the N-terminal tails of histone proteins. In general, acetylation marks are enriched in open chromatin regions, while histone methylation associates with either open or closed chromatin depending on the degree of methylation and the specific amino acid residues that are modified on the histone proteins (Suganuma and Workman, 2011). Changes in chromatin structure also occur through DNA methylation and demethylation, nucleosome repositioning and the generation of DNase hypersensitive sites. Conclusive evidence has demonstrated that epigenetic changes that take place during cell differentiation can persist through multiple cell divisions (Greer and Shi, 2012).

Natural killer (NK) cells constitute an arm of the innate immune system, as their effector functions are controlled by a repertoire of germline–encoded receptors that do not undergo somatic recombination (Lanier, 2008; Bryceson et al., 2011). However, similar to other lymphocyte subsets such as T and B cells, NK cells may manifest adaptive features including enhanced longevity and recall responses (Sun and Lanier, 2011). NK cells produce interferon (IFN)-γ in response to exogenous cytokines, display immunoregulatory activity in regards to perforin-dependent killing of activated immune cells and mediate immunosurveillance and elimination of intracellular pathogens and tumors through both IFN-γ secretion and target cell killing (Lodoen and Lanier, 2006; Vesely et al., 2011). From a clinical perspective, NK cells can kill allogeneic cells in the setting of hematopoietic stem cell transplantation and are utilized in immunotherapy for the treatment of certain malignancies (Geller and Miller, 2011).

**Figure 1:**
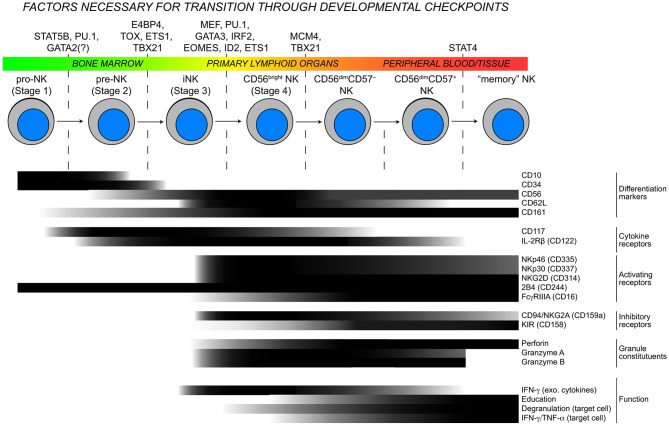
**Checkpoints in NK cell development.** As NK cells develop, they transit through the body and advance through checkpoints that are controlled by DNA-binding factors. Throughout this process, the expression of developmental markers, cytokine receptors, natural cytotoxicity receptors, and functional competencies are all dynamically regulated resulting in considerable heterogeneity within the NK cell population. Protein expression data are modified from (Freud et al., 2005; Freud and Caligiuri, 2006) and for “memory” NK cells extrapolated from (Sun et al., 2009; Hwang et al., 2012).

Considerable heterogeneity exists among human peripheral blood NK cells with regards to phenotype and function, and a hierarchy of activating stimuli controls the induction of various effector functions (Bjorkstrom et al., 2010; Fauriat et al., 2010; Juelke et al., 2010) (Figure 1). Although many details are lacking, experimental evidence suggests that NK cell precursors develop in primary lymphoid organs, circulate through the bloodstream, and collect in secondary lymphoid tissues. During the maturation process, NK cells differentiate into highly proliferative CD56^bright^ NK cells. Terminal differentiation involves a downregulation of CD56, changes in the receptor profile and acquisition of cytotoxic function (Freud et al., 2005). In parallel to the continuous maturation process, recognition of major histocompatibility complex (MHC) class I molecules by inhibitory receptors potentiate NK cell responses through a phenomenon known as education (Kim et al., 2005; Anfossi et al., 2006). The expression levels of several surface proteins on CD56^dim^ NK cells including CD57, CD62L, and CD94 correlate either positively or negatively with differentiation as defined by cytotoxic potential (Bjorkstrom et al., 2010; Juelke et al., 2010; Yu et al., 2010), but no cellular marker has been identified to distinguish between educated and non-educated NK cells. Further highlighting cellular plasticity, phenotypically and functionally distinct NK cell subsets are found in the uterus, liver, mucosa, and lymph nodes (Koopman et al., 2003; Cella et al., 2009; Luci et al., 2009). Defining NK cell subsets and understanding the acquisition of function is particularly difficult since superimposing differentiation and education processes on NK cell functionality does not fully explain the heterogeneity in NK cell responses. Here, we provide an overview of current insights into the transcriptional and epigenetic regulation of mature NK cell development and differentiation.

## Transcription factors implicated in development and differentiation of mature NK cells

Cellular identity is formed and often maintained by a network of transcription factors that influence the chromatin state and drive gene expression patterns. Mouse models have been instrumental for identifying several key transcription factors that are necessary for commitment to the NK cell lineage. Signaling through the interleukin-15 receptor common beta and gamma chains (CD122/132) is essential for NK cell development, and conditional knockout of *Stat5a* and *Stat5b* in NK cells results in an almost complete developmental block at the NK progenitor (NKP) to immature NK (iNK) transition (Eckelhart et al., 2011). Moreover, autosomal recessive *STAT5B* mutations in humans are associated with defective NK cell development and severe herpes virus infections (Kofoed et al., 2003). STAT5 proteins can enhance cell survival by driving expression of important anti-apoptotic genes such as *Bcl-2*, *Bcl-x*_*L*_, and *Mcl-1* (Debierre-Grockiego, 2004) or by inducing activation of the phosphoinostol 3-kinase (PI3K)/Akt and Ras/MAPK pathways (Nyga et al., 2005). While these pathways may contribute to NK cell development, Gascoyne *et al*. recently discovered an axis in which IL-15R signaling is necessary, specifically in NK cells, for the expression of the transcription factor E4bp4 (Gascoyne et al., 2009). As NKP cells transition to the iNK stage, E4bp4 induces transcription of *Id2* and *Gata3*, which are critical for further NK cell development. The generation of mice with targeted mutations in putative Stat5 binding sites within the regulatory regions *E4bp4* are needed to definitively determine whether the requirement for Stat5 proteins in NK cell development is through direct induction of *E4bp4* expression. A second axis involved in the control of early NK cell development is dependent upon the transcription factor thymocyte selection-associated high-mobility group box protein (TOX). NK cells in *Tox*-deficient mice fail to develop from iNK to mature NK (mNK) cells (Aliahmad et al., 2010). Although Id2 expression is slightly lower in *Tox*-deficient NK cells, overexpression of Id2 in these cells cannot rescue the developmental block. *Tox*-deficient NKPs also express normal levels of IL-15R, suggesting that TOX acts in a separate pathway to promote early NK cell development.

The homologous transcription factors Tbx21 (T-bet) and Eomes are essential components of another early pathway that regulates NK cell development. NK cell-specific deletion of *Tbx21* leads to a block in development at the transition between the NKP and iNK stages of development, while deletion of *Eomes* causes defects in subsequent maturation events including the acquisition of a diverse Ly49 receptor repertoire and expression of other developmental markers including DX5. Interestingly, sustained expression of both T-bet and Eomes appears to be essential for maintaining the identity/maturity of NK cells (Gordon et al., 2012), though the epigenetic basis for these observations is yet to be explored. Knockout studies in mice have demonstrated that several other transcription factors including *Ets1*, *Spi1*, and *Irf2* have cell-intrinsic requirements for early NK cell development (Scott et al., 1994; Barton et al., 1998; Lohoff et al., 2000). *Spi1*^−/−^ mice, which lack expression of the Ets-family transcription factor PU.1, die *in utero*. Fetal liver transfer experiments have revealed that PU.1-deficiency abrogates T and B cell development and severely impairs the development of NK cell precursors (pre-NK) (Colucci et al., 2001). Similarly, *Gata3*^−/−^ mice die *in utero*, but display normal NK cell numbers in the spleen. *Gata3*^−/−^ NK cells are more immature, suggesting a defect in iNK to mNK transition (Samson et al., 2003). Furthermore, *Id2*^−/−^ mice are viable and display a defect in iNK to mNK transition (Boos et al., 2007). Mutations in *GATA2* were recently identified in patients with a syndrome known as dendritic cell, monocyte, B and natural killer lymphoid (DCML)-deficiency (Dickinson et al., 2011; Hsu et al., 2011), but whether NK cells have a cell-intrinsic requirement for GATA2 expression or whether the NK cell-deficiency is secondary to the loss of monocytes and dendritic cells still needs to be clarified.

While considerable progress has been made in identifying transcription factor networks that control early developmental checkpoints, much less is known with regards to how the terminal maturation stages are regulated in NK cells. Targeted exome sequencing of individuals with a variant of familial glucocorticoid deficiency (FGD) associated with severe herpes virus infections uncovered an essential role for the minichromosome maintenance (MCM) 4 gene NK cell terminal differentiation (Gineau et al., 2012). MCM4 is a component of a protein complex with DNA helicase activity that acts in separating DNA strands during replication. NK cells from patients with partial MCM4-deficiency failed to efficiently differentiate beyond the CD56^bright^ stage and did not proliferate in response to IL-2 or IL-15, suggesting that robust proliferation is necessary for the CD56^bright^ to CD56^dim^ NK cell transition. It is remarkable that the proliferation of B and T cells was not affected in patients with MCM4-deficiency, and more studies are required to explain why the observed proliferation defects are specific to NK cells, whether MCM4-deficiency affects differentiation or survival and to rule out the possibility that the adrenal insufficiency in these patients is impacting NK cell development. Regardless of the mechanistic details, these studies highlight the uniqueness of the CD56^bright^ NK cell subset and demonstrate the importance of proliferation in NK cell development and differentiation.

## Evidence for epigenetic regulation of mature NK cell function

Although NK cell activation has been studied intensively for several decades, the mechanisms underlying the generation and maintenance of functional NK cells remains only partially understood. The effector molecule that is most highly associated with NK cell cytotoxicity is perforin, which creates pores in the phospholipid bilayer of target cells, facilitating entry of apoptosis-inducing granzymes. Expression of the Ets family transcription factor myeloid Elf1-like factor (MEF) directly binds to two sites within the *PRF1* promoter and is obligatory for perforin expression in NK cells but not CD8^+^ T cells, demonstrating that *PRF1* expression is differentially regulated at the transcriptional level in cytotoxic lymphocytes. The 5′ regulatory region of *PRF1* contains two enhancers, located at −15 and −1 kb that bind Stat5 and are responsive to IL-2R-activated signal transduction (Zhang et al., 1999). The importance of these enhancers is evident in NK cells from *Il2Rb* or *Stat5b* knockout mice that have significantly lower levels of perforin transcription (Imada et al., 1998). Interestingly, the −1 kb enhancer is also responsive to IL-6 and IL-12 and can bind STAT1a and STAT4, respectively, upon cytokine stimulation (Yu et al., 1999; Yamamoto et al., 2002). How these enhancers and other known regulatory elements within *PRF1* are regulated during NK cell development and whether they contribute to DNA demethylation within the locus remains to be determined.

Granzymes constitute a family of serine proteases that are found in cytolytic granules of cytotoxic T cells and NK cells. One of the most thoroughly studied members of the family is granzyme B, which can mediate target cell death through the cleavage of caspases or through damage to the mitochondria (Lieberman, 2003). Studies on transcriptional control of granzyme B expression in CD8^+^ T cells have demonstrated that several transcription factors including AP1, IKAROS, RUNX1, CREB1, and Eomes bind within a large DNase-hypersensitive region comprising the granzyme B promoter and facilitate transcription (Wargnier et al., 1995; Babichuk et al., 1996; Araki et al., 2008). Little is known with regards to epigenetic control of granzyme B expression outside of the observation that histone H3K9 acetylation within the granzyme B promoter correlates with high levels of gene expression (Araki et al., 2008). Signaling through the NF-κ B pathway induces granzyme B expression, and NF-κ B binds to a putative enhancer element approximately 10 kb downstream of the transcriptional start site (Huang et al., 2006). Further studies are needed to test whether this enhancer promotes expression by influencing the chromatin environment surrounding *GZMB*.

The mechanisms that regulate IFN-γ production are of interest given that a tight balance must be maintained to ensure that enough IFN-γ is produced to bolster immune responses without an overproduction that can lead to severe and widespread pathology. In this regard, CD56^bright^ NK cells produce IFN-γ mainly in response to exogenous cytokines, whereas CD56^dim^ NK cells do so mainly in response to target cells. Furthermore, IFN-γ production by donor NK cells is also impaired following transplantation (Foley et al., 2011). Rapid production of IFN-γ by NK cells upon stimulation appears to be due, at least in part, to the establishment of long-range histone acetylation patterns at distal conserved non-coding sequences (CNS) within the *IFNG* locus early during NK cell development. Several CNS elements have been identified in both the human and mouse genes. Of note, a CNS element ~22 kb upstream of murine *Ifng* binds T-bet and serves as an obligate enhancer for opening the locus and allowing expression in NK cells (Hatton et al., 2006).

The binding of several cytokines, including IL-2, IL-15, IL-12, and IL-18, to their cognate receptor complexes on NK cells strongly induces IFN-γ expression. In primary human NK cells, IL-2 treatment enhances histone acetylation at a DNase-I hypersensitivity region containing a Stat5 binding site ~3.5–4.0 kb upstream of the transcriptional start site. Thus, this distal regulatory element is a target of chromatin remodeling in the *IFNG* locus and acts as an IL-2-responsive transcriptional enhancer (Bream et al., 2004). Since CD56^bright^ NK cells constitutively express both the high- and intermediate-affinity IL-2R chains (Matos et al., 1993), the presence of the ~3.5–4.0 kb upstream element provides a presumptive explanation for the enhanced ability of CD56^bright^ NK cells to produce IFN-γ in response to IL-2 relative to the CD56^dim^ subset.

Changes in nucleosome composition or position are also important for the establishment of open chromatin and active transcription. So far, three distinct chromatin-remodeling complexes have been identified in mammals. These include the Mi-2-NuRD complex, the ISWI complex, and a mammalian Switch (Swi)-sucrose non-fermenter (SNF) homologue complex (Narlikar et al., 2002). The mammalian Swi-SNF complex contains a homologue of Brahma-related gene 1 (Brg1) or Brahma and includes Brahma/Brg1-associated factors that are capable of binding DNA (Khavari et al., 1993). In Th1 cells, Stat4-dependent recruitment of Brg1 is necessary for nucleosome remodeling within the first 600 bases of the *Ifng* promoter (Zhang and Boothby, 2006). Whether Stat4 recruits Brg1 through a direct interaction or indirectly by promoting histone acetylation and creating favorable conditions for Brg1 binding is still unknown. A similar mechanism presumably controls IFN-γ expression in CD56^dim^ NK cells, which express high levels of both Brg1 and STAT4 (Cichocki and Bryceson, unpublished observations). How the *IFNG* locus may be distinctly regulated in CD56^bright^ and CD56^dim^ NK cells to control IFN-γ production induced by exogenous cytokines or target cell recognition may provide interesting insights into the mechanisms regulating functional specialization of NK cell subsets.

**Figure 2:**
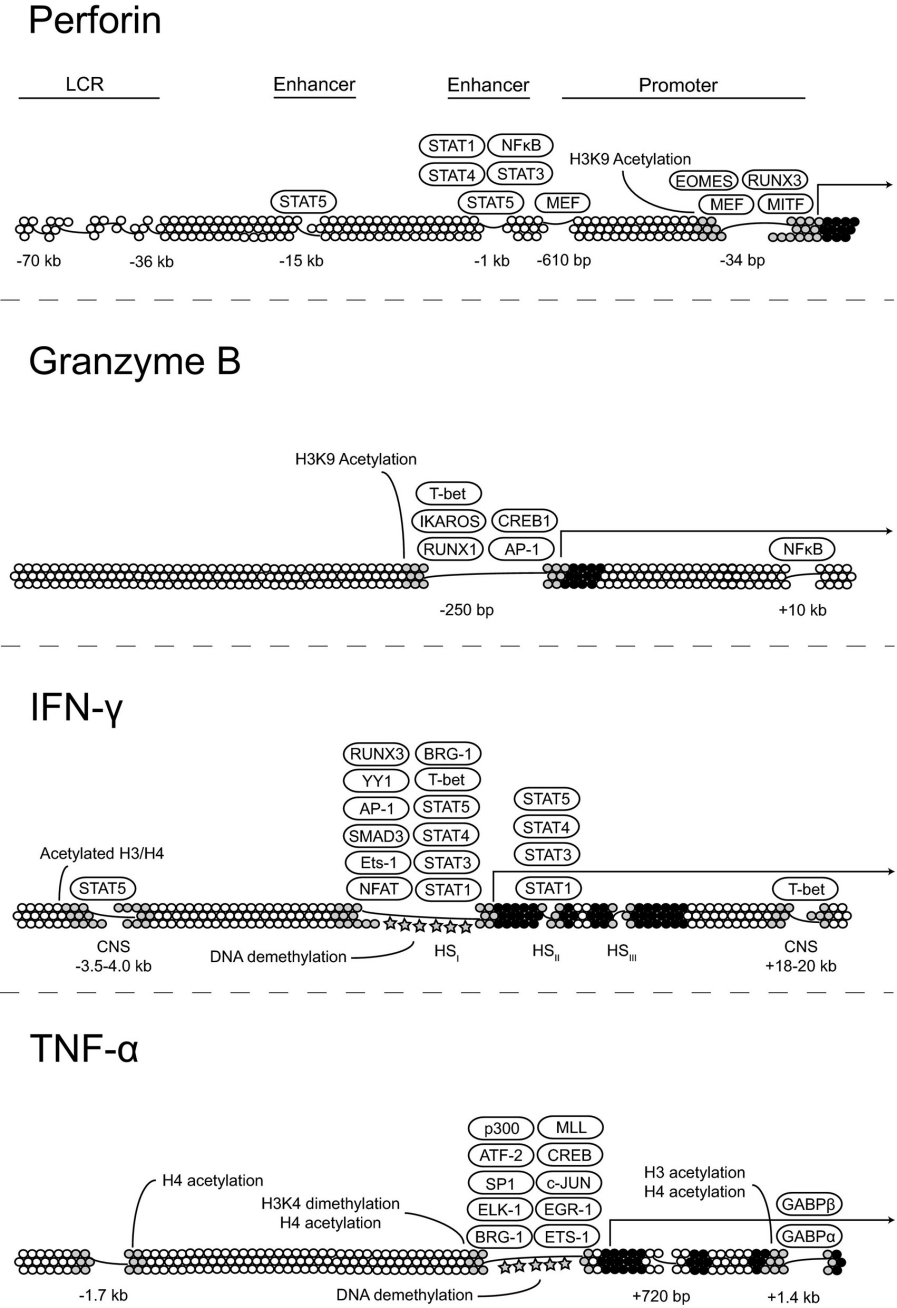
**Transcriptional and epigenetic regulation of key effector molecules.** Proposed models for the regulation of perforin, granzyme B, IFN-γ, and TNF-α expression at the transcriptional level through transcription factor binding and changes in the chromatin state.

In addition to IFN-γ, CD56^dim^ NK cells produce high levels of tumor necrosis factor (TNF)-α in response to target cell stimulation, which has a major role in initiating inflammatory responses, promoting the clearance of intracellular pathogens, and killing tumors. TNF-α belongs to the TNF superfamily, which also includes the proinflammatory cytokines lymphotoxin (LT)-α and LT-β. The *TNF/LT* genes are clustered together within the MHC class III region on human chromosome 6. DNA within the 5′ regulatory region of *TNF* is densely methylated in embryonic and hematopoietic precursor cells. Studies using monocytes show that lineage commitment is associated with DNA demethylation followed by the acquisition of histone acetylation and the establishment of open chromatin. In mouse monocyte cell lines, histones within the *Tnf* promoter associate constitutively with Brg1 (Ramirez-Carrozzi et al., 2006). Therefore, a common mechanism involving histone modification and nucleosome repositioning is responsible for creating an open chromatin conformation within the regulatory regions of multiple inflammatory cytokine genes. Figure 2 summarizes the current state of knowledge with regard to transcription factor binding to *PRF1*, *GZMB*, *IFNG*, and *TNF* and epigenetic control of their expression.

## Adaptive NK cell responses and epigenetic regulation

The term “memory” NK cell was first coined after the observation of long-lived NK cells following CMV infection in mice (Sun et al., 2009). Such adaptive NK cells express self-specific inhibitory Ly49 receptors and their expansion is *Dap12*-dependent (Sun et al., 2009). Similarly, in humans, NK cells expressing the DAP12-coupled activating receptor NKG2C are found at high frequencies in CMV seropositive donors (Lopez-Vergès et al., 2011; Foley et al., 2012). Moreover, in the context of severe viral infections, NKG2C^+^ NK cells with self-specific inhibitory KIR receptors expand (Bjorkstrom et al., 2011; Beziat et al., 2012).

The formation of B and T cell memory involves antigen recognition by the B cell receptor and T cell receptor complexes, respectively, along with cytokine signaling and clonal expansion. At the molecular level, programming of B and T cell memory is dependent upon transcription factor networks that promote differentiation and survival. Of note, the transcription factors B cell lymphoma 6 (BCL-6) and PR domain-containing protein 1 (PRDM1) act within a regulatory axis to control effector and memory differentiation in CD4^+^ T cells, CD8^+^ T cells, and B cells by reinforcing cell-fate decisions (Crotty et al., 2010). The shared requirement for BCL-6 and PRDM1 in the generation of adaptive immune memory make these transcription factors attractive candidates for study in the emerging field of NK cell memory formation.

**Figure 3:**
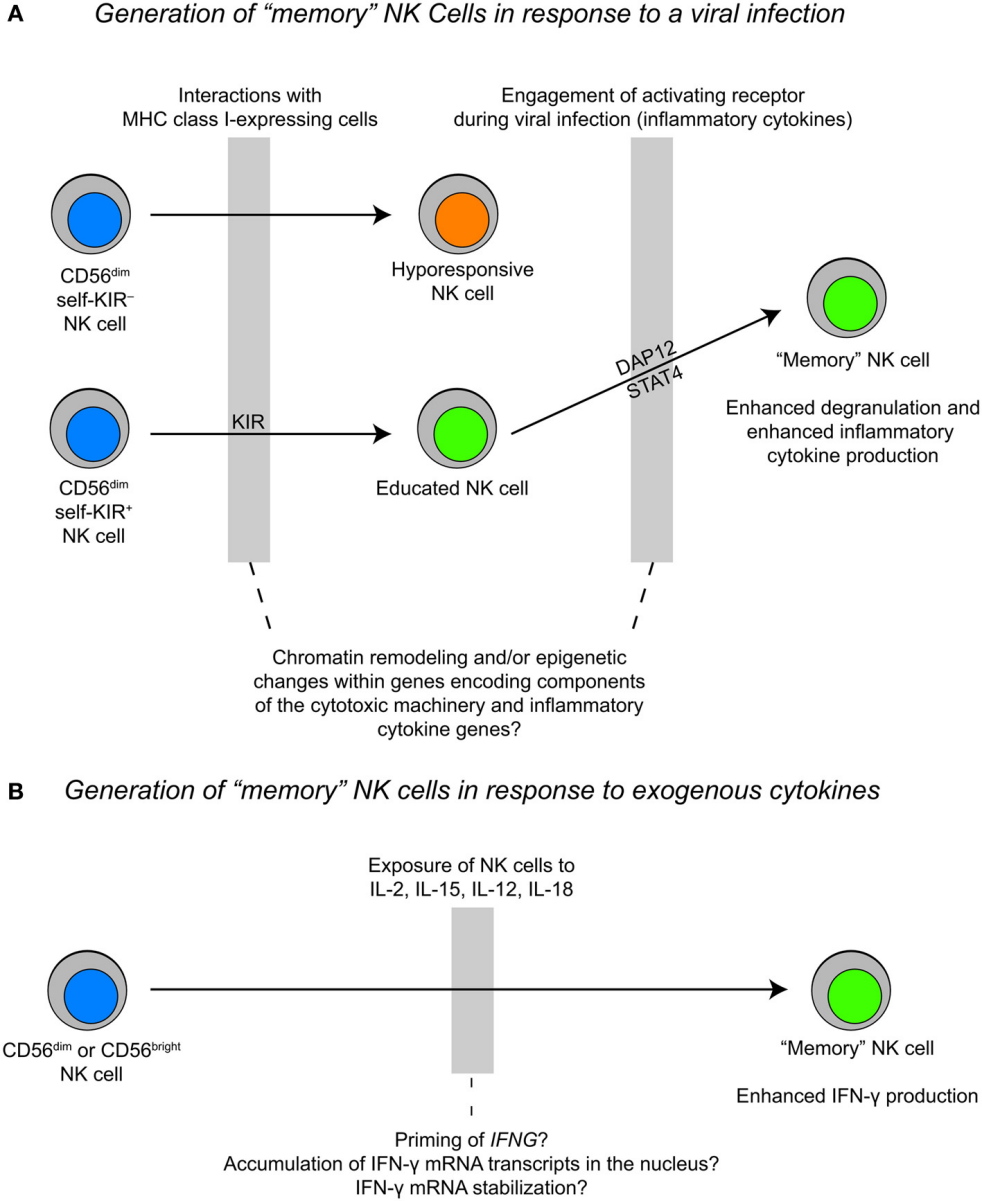
**Proposed models for the generation of “memory” NK cells during a viral infection vs. cytokine priming. (A)** The generation of “memory” NK cells during a viral infection likely happens within the context of NK cell education, where an “educated” NK cell expands during a viral infection and exhibits rapid cytotoxicity and inflammatory cytokine production upon a secondary challenge. **(B)** The generation of “memory” NK cells as a result of cytokine stimulation may result in epigenetic priming of the *IFNG* locus and/or accumulation of IFN-γ transcripts in the nucleus. This process may be fundamentally different from the generation of “memory” NK cells during a viral infection.

The mechanisms that control NK cell proliferation and survival are highly relevant to “memory” NK cells. Studies of murine NK cell responses to mouse cytomegalovirus (MCMV) revealed that virus-specific NK cells proliferate 100-fold in the spleen and 1000-fold in the liver after infection. After the initial expansion and contraction phases, a pool of self-renewing “memory” NK cells with the ability to rapidly respond to secondary viral challenges persists for several months (Sun et al., 2009). This adaptive NK cell population can develop in *Ifng*-deficient mice, but fails to develop in either *Il12r* or *Stat4*-deficient mice (Sun et al., 2012). The precise role of STAT4 in the generation of “memory” NK cells remains to be elucidated, but studies of memory formation in CD8^+^ T cells may provide some clues. STAT4 activation downstream of IL-12 promotes clonal expansion of antigen-specific CD8^+^ T cells by enhancing expression of the anti-apoptotic genes *Bcl2* and *Bcl-x*_*L*_ and increasing sensitivity to IL-7 and IL-15 (Li et al., 2006). Moreover, “memory” NK cells in mice vigorously produce IFN-γ and TNF-α in response to ITAM-receptor stimulation. The same phenomenon is observed in a subset of human NK cells with down-regulated Fcε Rγ-expression (Hwang et al., 2012). At present, data suggest that only educated cells proliferate in response to cytokines and signals from DAP12-coupled receptors (Figure 3). However, many open questions remain with respect to how NK cell function relates to development and the extent to which NK cell differentiation is linear vs. branched. The application of high-throughput sequencing technologies to study distinct subsets of NK cells could be very useful in elucidating the processes that regulate differentiation and the acquisition of function.

Also pertaining to stimulation induced changes in NK cell responsiveness, two recent reports described the generation of “memory” NK cells after *in vitro* stimulation with combinations of IL-12, IL-18, and IL-15 (Cooper et al., 2009; Romee et al., 2012). In both mouse and human systems, NK cells were primed with cytokines and restimulated at later time points. Cells that were primed demonstrated a more robust IFN-γ response without any enhancement in cytotoxicity upon restimulation with either exogenous cytokines or through activating receptors. These adaptive responses were identified primarily in human CD56^bright^ NK cells and CD56^dim^ NK cells lacking the expression of killer immunoglobulin-like receptors (KIR) and CD57, and might simply reflect epigenetic changes at the *IFNG* locus. An alternative explanation for these results is that the priming of NK cells with IL-12, IL-18, and IL-15 selectively affects the post-transcriptional regulation of IFN-γ mRNA. Previous studies have shown that co-stimulation of NK cells with IL-2 and IL-12 leads to enhanced mRNA stabilization (Ye et al., 1995). However, IL-12 stimulation is unique in that it causes an elevation in nuclear IFN-γ mRNA retention after transcriptional activity of the gene has ceased, which is likely directed by specific sequence elements within the IFN-γ mRNA transcript. The nuclear retention of IFN-γ mRNA transcripts can be overcome by co-stimulation with IL-2, which leads to transcription-independent transport of IFN-γ mRNA out of the nucleus and into the cytoplasm where it can be translated (Hodge et al., 2002). Enhanced cytokine responses in memory T cells correlate with changes in the chromatin state of cytokine genes. These changes are stable and can be inherited through cell divisions, forming the basis for memory. Future studies should be done to compare the epigenetic changes associated with “memory” NK cells generated from cytokine priming to “memory” NK cells generated from receptor engagement during a viral infection, which exhibit more robust IFN-γ responses and enhanced degranulation upon secondary challenge.

## Perspectives and future directions

A deeper understanding of NK cell differentiation and the specialization of function will ultimately require high-resolution, genome-wide analyses. The development of the chromatin immunoprecipitation (ChIP)-seq technique, which combines ChIP and high-throughput sequencing methods, provides the opportunity to directly assess histone modifications and DNA-binding proteins. Combining ChIP-seq data with global gene expression results from techniques such as microarrays or RNA-seq makes it possible to produce a genome-wide view that links chromatin state with transcriptional activity within a defined population of cells. These technologies have been used extensively to study T cell differentiation and the chromatin states of genes that are selectively expressed in distinct populations of memory cells. Limited numbers relative to other lymphocyte subsets makes the application of high-throughput technologies to primary NK cells difficult, but improvements in reagents and sequencing sensitivity should soon overcome these obstacles. This will provide new views of how NK cells differentiate and undergo functional specialization.

### Conflict of interest statement

The authors declare that the research was conducted in the absence of any commercial or financial relationships that could be construed as a potential conflict of interest.
